# Supplementary material for: Shape-memory effect in twisted ferroic nanocomposites
Source: Nat Commun. 2023 Feb 10;14:750. doi: 10.1038/s41467-023-36274-w (PMC9918508; doi:10.1038/s41467-023-36274-w)
Supplement: Supplementary file 1 — Supplementary Information [file 41467_2023_36274_MOESM1_ESM.pdf]

Supplementary Materials for

Shape-memory effect in twisted ferroic nanocomposites

Donghoon Kim†, Minsoo Kim†, Steffen Reidt, Hyeon Han, Ali Baghizadeh, Peng Zeng, Hongsoo Choi, Josep Puigmartí-Luis, Morgan Trassin, Bradley J. Nelson, Xiang-Zhong Chen*, Salvador Pané*

Correspondence to: chenxian@ethz.ch, vidalp@ethz.ch

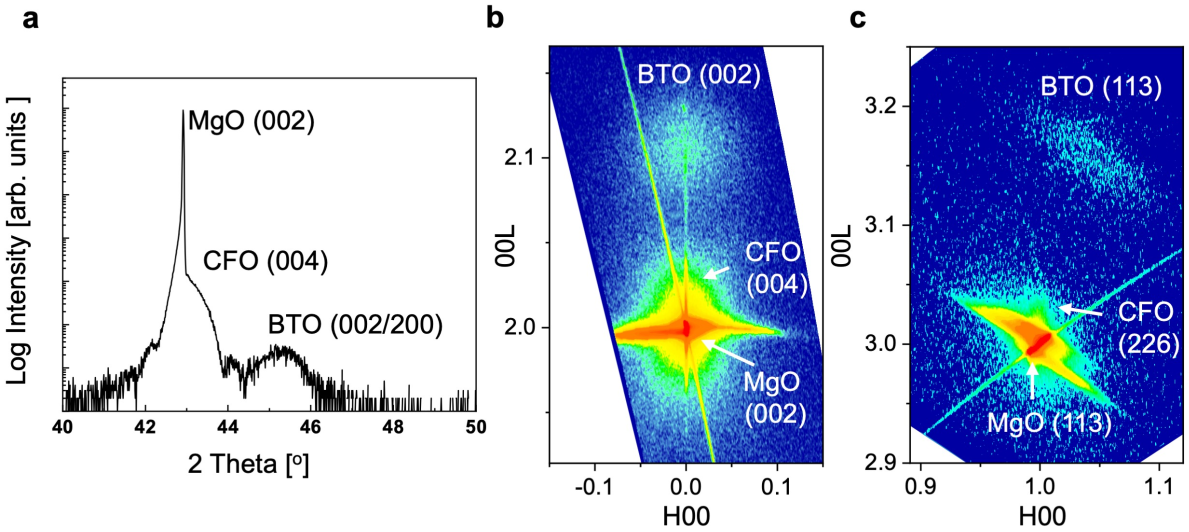

Fig. S1. Epitaxial growth of BTO/CFO bilayer thin films. (a) Theta-2theta scan of the BTO/CFO epitaxial thin film grown on MgO (001) substrate. (b-c) Reciprocal Space Mappings of the BTO/CFO//MgO thin film around MgO (002) and (113) Bragg peaks show epitaxial characteristics of the bilayer thin film with the relaxed strain state of the BTO layer.

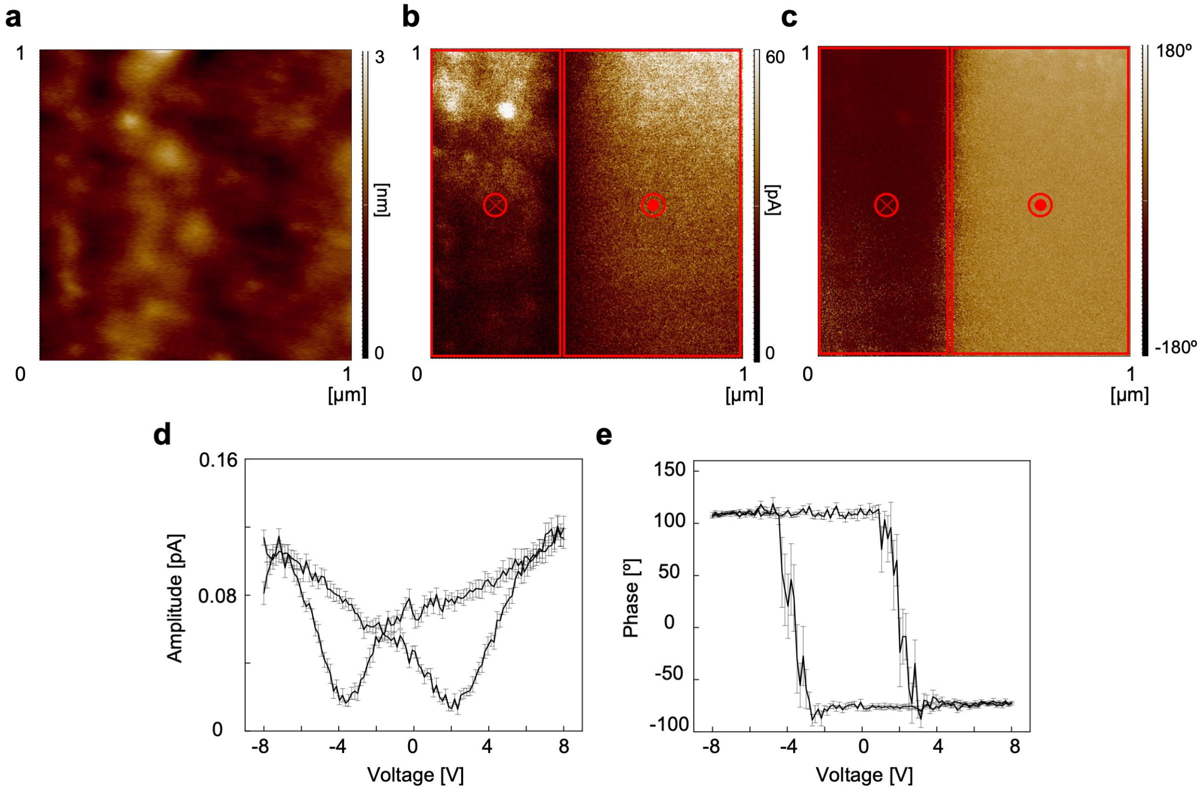

Fig. S2. Ferroelectric properties of free-standing BTO/CFO membranes. Ferroelectric properties were confirmed using piezoresponse force microscope (PFM). PFM (a) topography, (b) amplitude, and (c) phase images and local piezoelectric hysteresis (d) amplitude and (e) phase loops clearly show ferroelectric domain switching properties of BTO layer in BTO/CFO. Error bars in (d) and (e) indicate the standard deviations among 5 consecutive measurements.

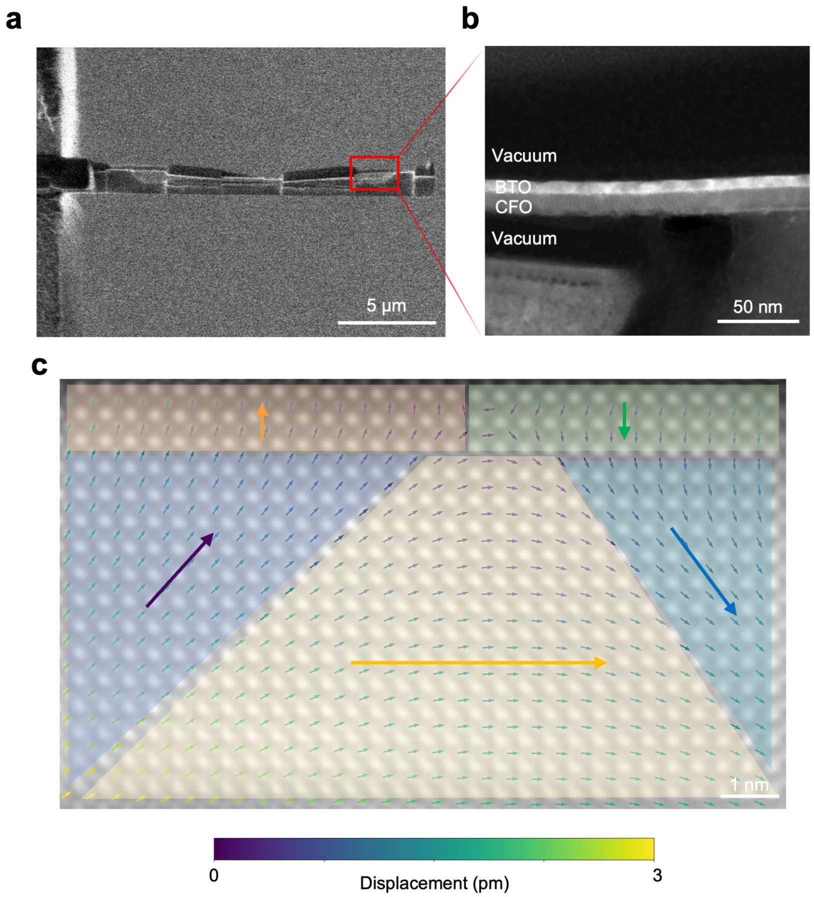

Fig. S3. High-angle annular dark field scanning transmission electron microscope (HAADF-STEM) analysis of free-standing BTO/CFO membrane. (a) Focused ion beam (FIB) lamella of the released BTO/CFO membrane. (b) HAADF-STEM image of the free-standing BTO/CFO layer. (c) Ti-ion displacements have been mapped onto the HAADF-STEM images. In-plane ferroelectric polarization domains and the rotation of the polarization were observed in the BTO layer.

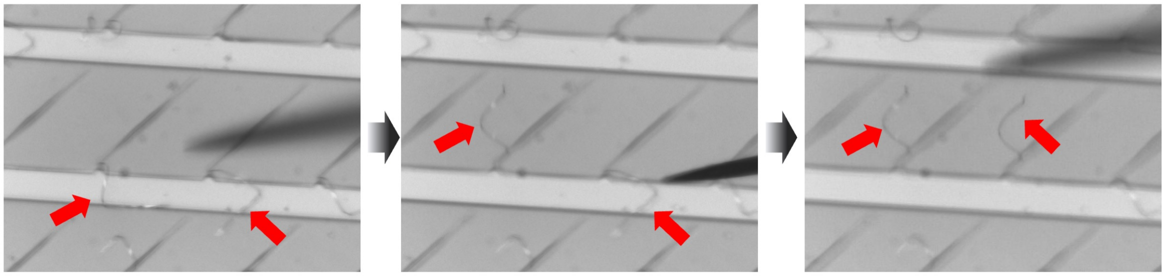

Fig. S4. Superelasticity of twisted BTO/CFO nanocomposites. Full shape recovery of the distorted structures into twisted architectures when detached from the substrate. A full video of the detachment and the pulling and stretching process is available in Movie S1.

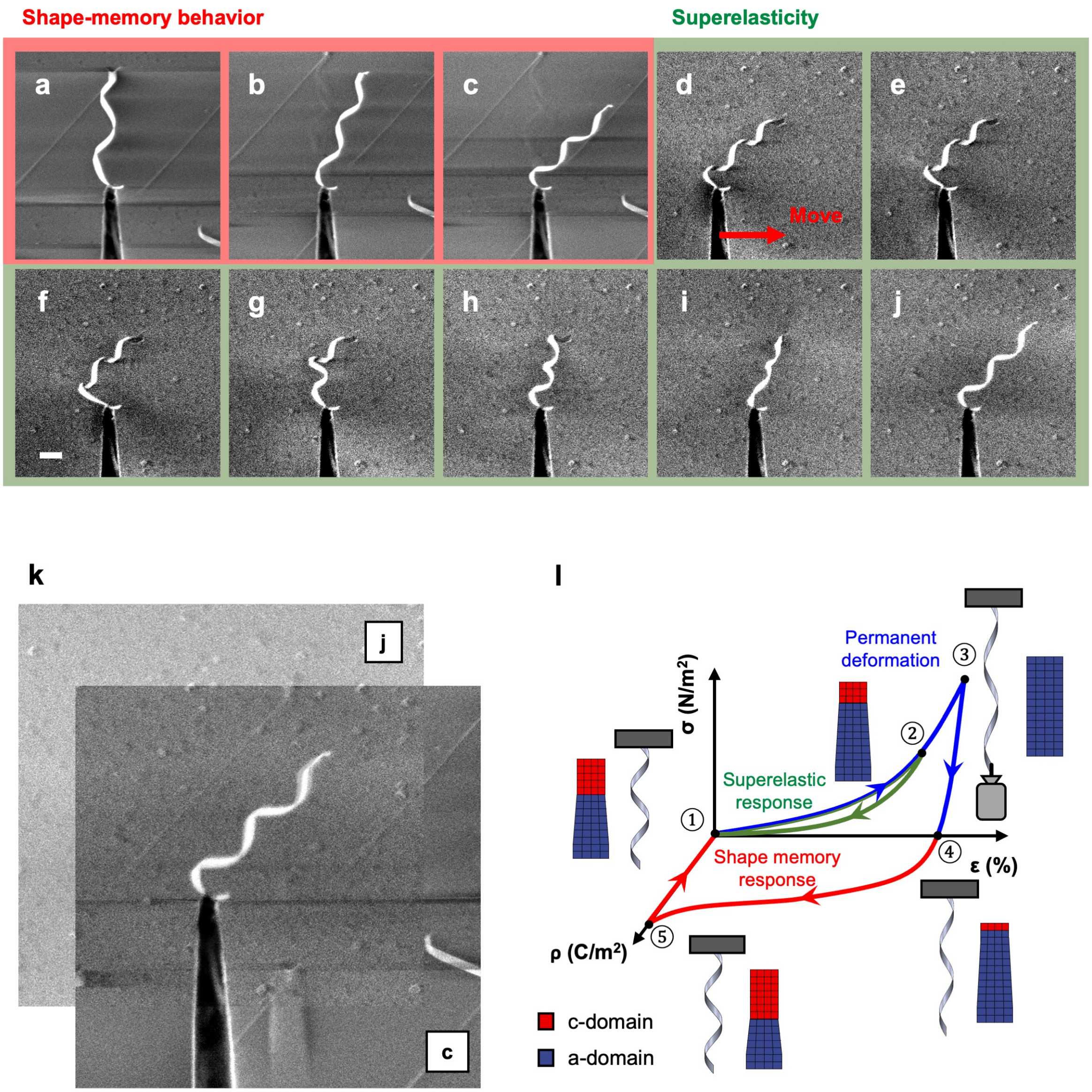

Fig. S5. Maintaining superelasticity after showing shape-memory response under electron beam irradiation. (a) The structure was stretched with the force sensor probe (1→2). (b) When enough tensile load was applied, the twisted BTO/CFO was fractured and the deformation was maintained (3→4). (c) Shape-memory effect when irradiated with electron beam (4→5→1). (d-i) Superelasticity of the recovered structure was tested using Van der Waals force between the substrate and the twisted BTO/CFO. Superelasticity was preserved after the shape memory behavior. (j) The shape was recovered after the electron beam irradiation. (k) Comparison of the physical shapes between (c) and (j) (before and after the superelasticity test), indicating full recovery of the twisted architecture. (l) Superelastic and shape-memory response cycle in twisted BTO/CFO with corresponding domain switching in the BTO layer.

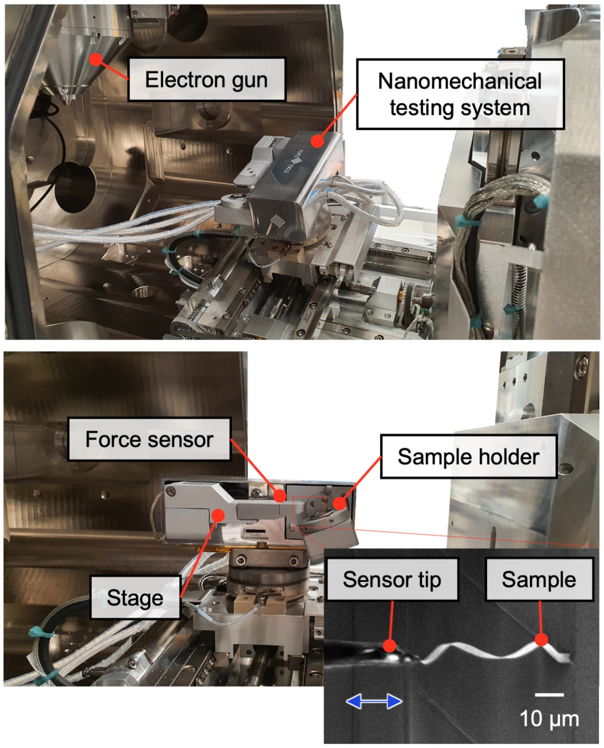

Fig. S6. *In-situ* nanomechanical measurement setup (FT-NMT03, Femtotools AG) in SEM.

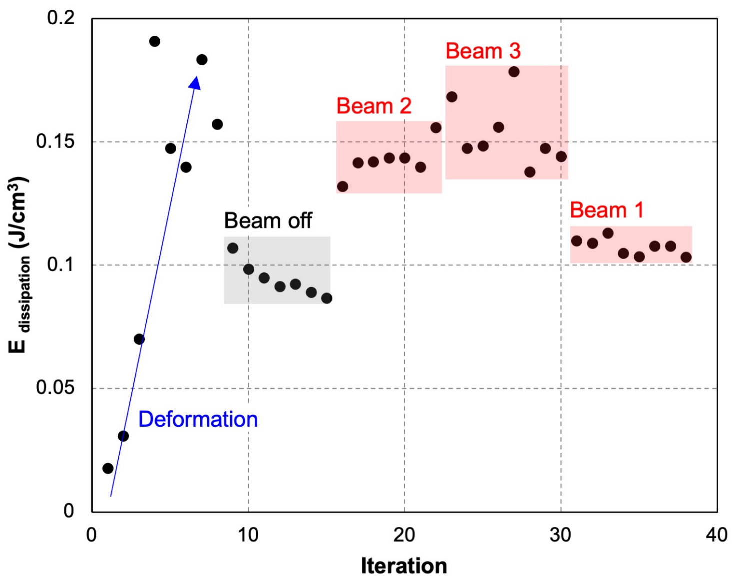

Fig. S7. Electron beam current (dose) dependence of the $E_{dissipation}$. Beam 1, 2, and 3 indicate 30 pA, 100 pA, and 400 pA beam current, respectively, while maintaining the same magnification. With a stronger electron beam current, higher energy dissipation was obtained.

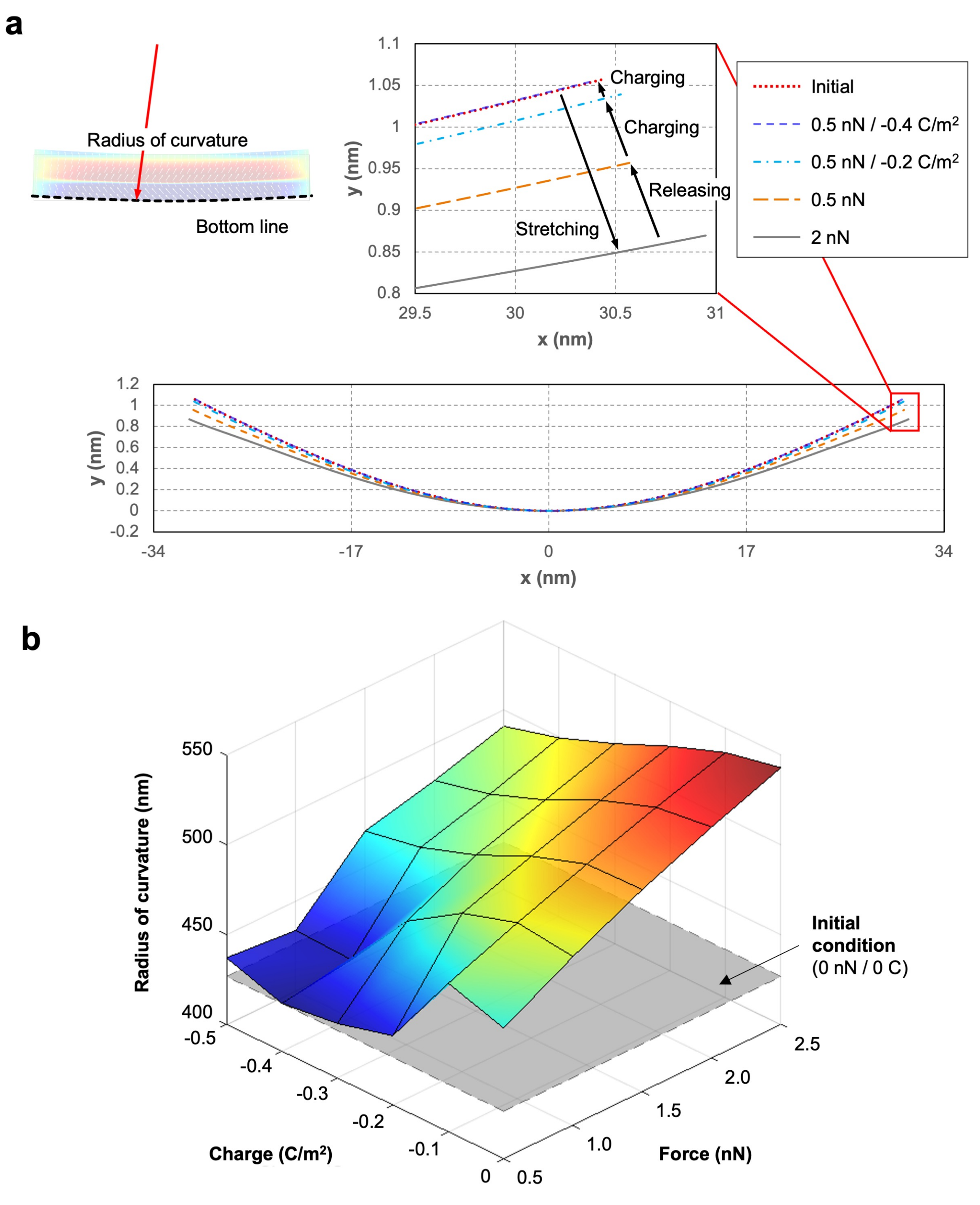

Fig. S8. The effect of the tensile force and surface charge accumulation on the physical shape of the BTO slab calculated with phase-field modelling. (a) Displacements of the bottom surface of the BTO slab are plotted under different tensile force and surface charge boundary conditions. The values of the tensile forces and surface charges were assumed to be in states of stretching, releasing, and charging. Higher tensile forces resulted in more stretching in the x-direction, while

surface charge accumulation resulted in a physical shape recovery to the initial state. Here, the initial state refers to the bent BTO slab, where the bottom surface was tensile-strained due to the lattice mismatch between the BTO and the CFO. (b) Calculated radius of curvature of the bottom surface as a function of surface charges and stretching forces. As more surface charges are accumulated, the radius of curvature value gets closer to the initial condition, indicating shape recovery to the original state.

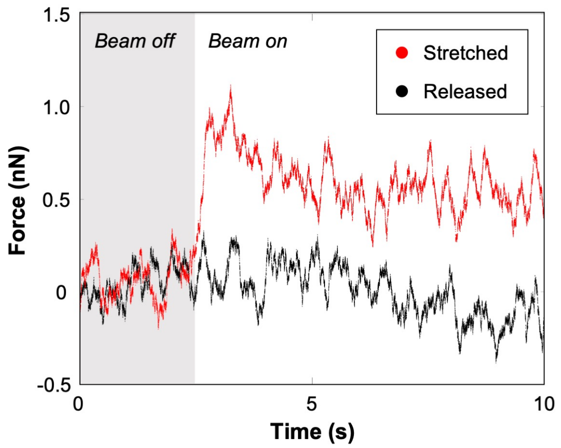

Fig. S9. Actuation force triggered by the electron beam irradiation. Actuation forces were measured by attaching nanomechanical force sensor at one end of the BTO/CFO twist. Black and red lines indicate forces measured as a function of time while the BTO/CFO twist was at the initial (released) state or at the stretched state, respectively. When the BTO/CFO twist was not stretched (black), there was no response when the electron beam was irradiated. However, when the twist was stretched, the actuation force was measured when electron beam was irradiated onto the structure. If there is any electrostatic charging effect, the actuation force should be observed in BTO/CFO twist whether the structure is stretched or not. The fact that the actuation force was only observed when the BTO/CFO twist was stretched show that the 'recovery' force is triggered only when there is a deformation. Therefore, the electrostatic charging effect can be ruled out. The vibration effect can be excluded as well. If there is any vibration generated by the electron beam exposure, the actuation force should depend on the beam scanning time. However, we have not observed any scanning time dependency, suggesting that the vibration effect on the recovery of the $E_{dissipation}$ is negligible.

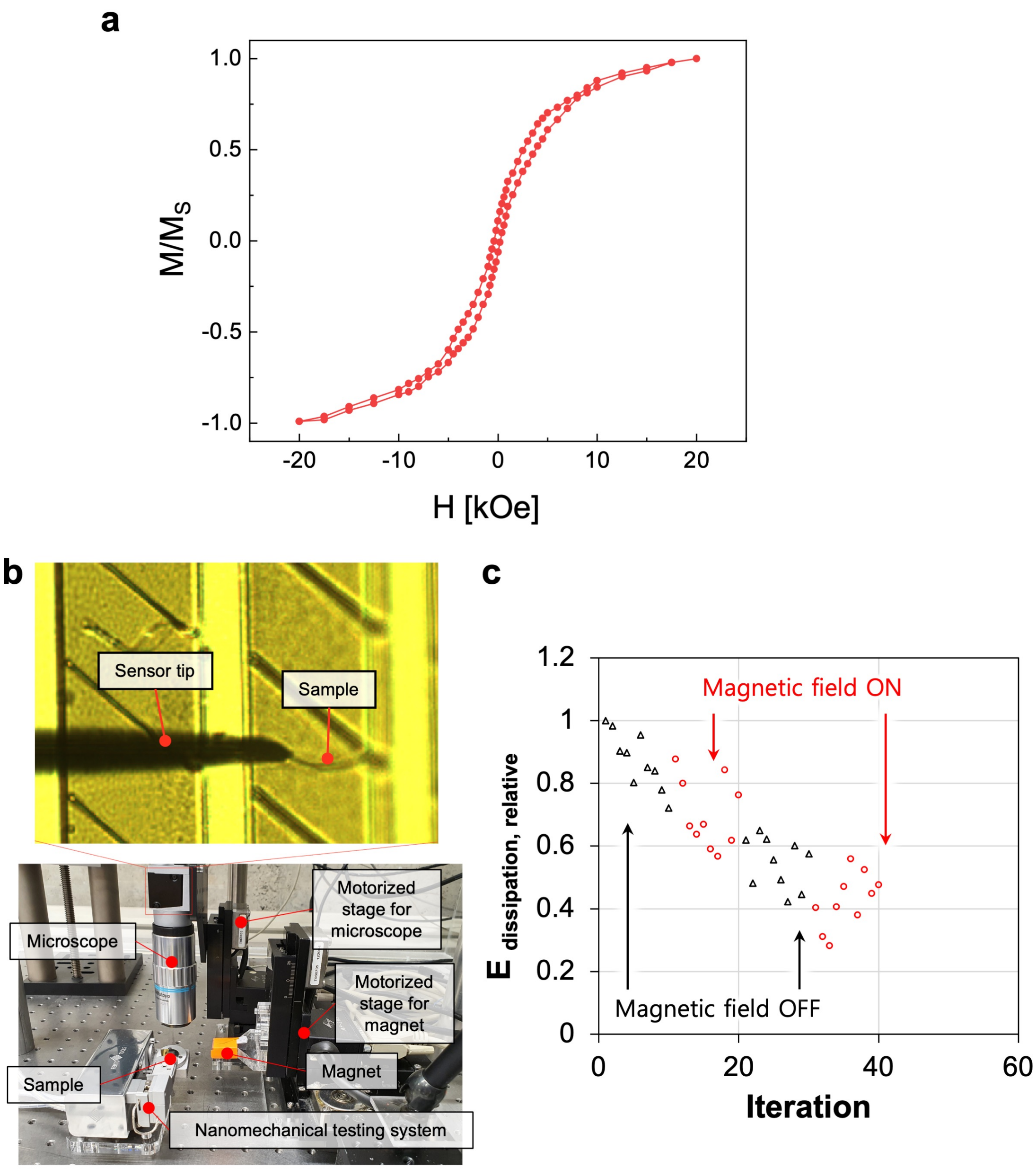

Fig. S10. Magnetic field dependency of the mechanical properties of BTO/CFO microhelices. (a) Magnetic hysteresis loop of the BTO/CFO//MgO (001) thin film. (b) *In-situ* nanomechanical measurement setup under optical microscope with magnetic field application system. The tensile test under optical microscope is available in Movie S6. Magnetic field was calibrated with magnetometer before applying the field. (c) Effect of the magnetic field (~50 mT) on the $E_{dissipation}$ over the tensile cycling tests. Unlike electron beam irradiation, no significant recovery was observed.

Movie S1.

Superelasticity test of BTO/CFO twisted architectures. Distorted structures recover their original shape right after being mechanically detached from the substrate. Even after the applications of pushing and pulling forces, the structure recovers its original twisted architecture.

Movie S2.

Electron beam induced shape-memory effect of BTO/CFO twisted architecture. After the application of the large tensile stress, the structure maintained the deformation. When the electron beam is focused on the deformed structures, it recovered its initial shape.

Movie S3.

In-situ nanomechanical tensile test. One edge of the twisted BTO/CFO was attached to the force sensor by SEM-compatible glue and force-displacement curve was measured during the tensile test.

Movie S4.

Second cycle of the shape-memory effect. After the first shape recovery, the twisted BTO/CFO was deformed again using Van der Waals force from the substrate. With the irradiation of the electron beam, deformed structure recovered the initial shape, showing repeated shape-memory effect.

Movie S5.

Third cycle of the shape-memory effect. After the second cycle, the structure was deformed using electrostatic force from the substrate. Again, with the irradiation of the electron beam, the structure recovered the initial shape.

Movie S6.

In-situ nanomechanical tensile test under an optical microscope for evaluation of magnetic field dependency.
