# Supplementary material for: Shape-memory effect in twisted ferroic nanocomposites
Source: Nat Commun. 2023 Feb 10;14:750. doi: 10.1038/s41467-023-36274-w (PMC9918508; doi:10.1038/s41467-023-36274-w)
Supplement: Supplementary file 3 — Description of Additional Supplementary Files [file 41467_2023_36274_MOESM3_ESM.pdf]

Description of Additional Supplementary Files

Supplementary Movie 1.

Superelasticity test of BTO/CFO twisted architectures. Distorted structures recover their original shape right after being mechanically detached from the substrate. Even after the applications of pushing and pulling forces, the structure recovers its original twisted architecture.

Supplementary Movie 2.

Electron beam induced shape-memory effect of BTO/CFO twisted architecture. After the application of the large tensile stress, the structure maintained the deformation. When the electron beam is focused on the deformed structures, it recovered its initial shape.

Supplementary Movie 3

In-situ nanomechanical tensile test. One edge of the twisted BTO/CFO was attached to the force sensor by SEM-compatible glue and force-displacement curve was measured during the tensile test.

Supplementary Movie 4

Second cycle of the shape-memory effect. After the first shape recovery, the twisted BTO/CFO was deformed again using Van der Waals force from the substrate. With the irradiation of the electron beam, deformed structure recovered the initial shape, showing repeated shape-memory effect.

Supplementary Movie 5

Third cycle of the shape-memory effect. After the second cycle, the structure was deformed using electrostatic force from the substrate. Again, with the irradiation of the electron beam, the structure recovered the initial shape.

Supplementary Movie 6

In-situ nanomechanical tensile test under an optical microscope for evaluation of magnetic field dependency.
