# Supplementary material for: Shape-memory effect in twisted ferroic nanocomposites
Source: Nat Commun. 2023 Feb 10;14:750. doi: 10.1038/s41467-023-36274-w (PMC9918508; doi:10.1038/s41467-023-36274-w)
Supplement: Supplementary file 10 — Lasing Reporting Summary [file 41467_2023_36274_MOESM10_ESM.pdf]

Corresponding author(s): Xiangzhong Chen

Lasing Reporting Summary

Nature Research wishes to improve the reproducibility of the work that we publish. This form is intended for publication with all accepted papers reporting claims of lasing and provides structure for consistency and transparency in reporting. Some list items might not apply to an individual manuscript, but all fields must be completed for clarity.

For further information on Nature Research policies, including our data availability policy, see Authors & Referees.

ü Experimental design

Please check: are the following details reported in the manuscript?

1. Threshold

   Plots of device output power versus pump power over a wide range of values indicating a clear threshold

   [ ] Yes [x] No — This item does not apply to our manuscript.

2. Linewidth narrowing

   Plots of spectral power density for the emission at pump powers below, around, and above the lasing threshold, indicating a clear linewidth narrowing at threshold

   [ ] Yes [x] No — This item does not apply to our manuscript.

   Resolution of the spectrometer used to make spectral measurements

   [ ] Yes [x] No — This item does not apply to our manuscript.

3. Coherent emission

   Measurements of the coherence and/or polarization of the emission

   [ ] Yes [x] No — This item does not apply to our manuscript.

4. Beam spatial profile

   Image and/or measurement of the spatial shape and profile of the emission, showing a well-defined beam above threshold

   [ ] Yes [x] No — This item does not apply to our manuscript.

5. Operating conditions

   Description of the laser and pumping conditions
   Continuous-wave, pulsed, temperature of operation

   [x] Yes [ ] No — The laser power and energy density have been calibrated before each deposition, and the values are described in the methods section.

   Threshold values provided as density values (e.g. W cm^{-2} or J cm^{-2}) taking into account the area of the device

   [ ] Yes [x] No — This item does not apply to our manuscript.

6. Alternative explanations

   Reasoning as to why alternative explanations have been ruled out as responsible for the emission characteristics
   e.g. amplified spontaneous, directional scattering; modification of fluorescence spectrum by the cavity

   [ ] Yes [x] No — This item does not apply to our manuscript.

7. Theoretical analysis

   Theoretical analysis that ensures that the experimental values measured are realistic and reasonable
   e.g. laser threshold, linewidth, cavity gain-loss, efficiency

   [ ] Yes [x] No — This item does not apply to our manuscript.

8. Statistics

   Number of devices fabricated and tested

   [ ] Yes [x] No — This item does not apply to our manuscript.

   Statistical analysis of the device performance and lifetime (time to failure)

   [ ] Yes [x] No — This item does not apply to our manuscript.
